# Antenatal visits are positively associated with uptake of tetanus toxoid and intermittent preventive treatment in pregnancy in Ivory Coast

**DOI:** 10.1186/s12889-019-7847-1

**Published:** 2019-11-06

**Authors:** Sanni Yaya, Komlan Kota, Amos Buh, Ghose Bishwajit

**Affiliations:** 1 School of International Development and Global Studies, Faculty of Social Sciences, University of Ottawa, 120, University Private, Ottawa, ON, Ottawa, ON K1N 6N5 Canada; 2 The George Institute for Global Health, The University of Oxford, Oxford, UK; 3 Interdisciplinary School of Health Sciences, Faculty of Health Sciences, University of Ottawa, Ottawa, Canada

**Keywords:** TT immunization, IPTp-SP drugs, Pregnant women, ANC visits, Ivory Coast

## Abstract

**Background:**

Malaria and tetanus infections among pregnant women represent two major public health problems in sub-Saharan Africa. Optimum use of Intermittent preventive treatment in pregnancy (IPTp) with sulfadoxine-pyrimethamine (IPTp-SP) and immunization against tetanus among pregnant women during antenatal care (ANC) visits are recommended strategies to prevent these issues. Despite these recommendations, many women in Africa remain deprived of these cost-effective and life-saving interventions. In this study, we aimed to examine the prevalence of women using these two services, and the association between women’s uptake of IPTp-SP and tetanus toxoid (TT) with antenatal care use in Ivory Coast.

**Methods:**

This study was based on the fifth round of Multiple Indicator Cluster Survey (MICS 5) conducted in Ivory Coast in 2016. Participants were 9583 women aged between 15 and 49 years. Outcomes were TT and Intermittent preventive treatment with sulfadoxine-pyrimethamine (IPTp-SP). Data analysis was conducted using bivariate and multiple logistic regression.

**Results:**

In this study, the prevalence of taking TT immunization and IPTp-SP drugs was 81.97 and 17.83% respectively. Of the participants who took these drugs at all, the prevalence of taking adequate doses of TT immunization was 78.75% and that of IPTp-SP was 35.46%. In the multivariable analysis model, higher age groups, 25–29 years (OR = 2.028, 95%CI = 1.120–3.669) were found to be positively associated with uptake of adequate doses of IPTp-SP drugs. Women who attended at least four ANC visits had higher odds of taking IPTp-SP drugs (OR = 1.656, 95%CI = 1.194–2.299) and TT immunization (OR = 2.347, 95%CI = 1.384–3.981), and also had higher odds of receiving adequate doses of IPTp-SP drugs (OR = 3.291, 95%CI = 2.157–5.020) and that of TT immunization (OR = 1.968, 95%CI = 1.398–2.771). The odds of taking IPTp-SP drugs were significantly higher among women with primary (OR = 2.504, 95%CI = 1.020–6.146) and secondary/higher education (OR = 3.298, 95%CI = 1.343–8.097) compared to those with no education. Also, women with higher parity had lower odds of taking TT immunization (OR = 0.218, 95%CI = 0.055–0.858) compared to those with lower parity. Findings from this study also revealed that the odds of taking adequate doses of IPTp-SP drugs were significantly lower among participants from Mandé du Nord ethnicity (OR = 0.378,95%CI = 0.145–0.983) compared to those from other ethnicities.

**Conclusion:**

In this study, uptake of IPTp-SP drugs was much lower than TT immunization. High number of ANC visits were found to be significantly associated with taking IPTp-SP drugs and TT immunization and also with that of taking them in adequate doses. Vaccination promotion is necessary to protect pregnant women and reduce adverse health outcomes among the newborn in Ivory Coast.

## Background

Tetanus and Malaria in pregnancy are a major public health problem causing maternal, fetal and infant morbidity and mortality especially in sub-Saharan Africa [1–4]. Human Malaria is caused by five different species of Plasmodium: *P. falciparum*, P. malariae, *P. ovale* and *P. vivax*, and transmitted to people though the bites of infected female Anopheles mosquitoes [5, 6] . In 2017, WHO estimated 219 million cases of malaria in 90 countries and 435,000 deaths related to malaria. The sub-Saharan African region has been reported to carry the highest proportion of the global malaria burden; accounting for 92% of all malaria cases and 93% of malaria deaths [7].

To prevent and reduce malaria transmission, two forms of vector control are recommended by WHO; sleeping under insecticide-treated bednets (ITNs) [8–10] and indoor residual spraying with insecticides [11–13]. Besides this, environmental management practices – clearing bushes and draining stagnant water around houses also provide a form of prevention [14–16]. To prevent malaria in pregnant women living in areas of moderate and high malaria transmission especially in Africa, intermittent preventive treatment in pregnancy (IPTp) with sulfadoxine pyrimethamine is recommended by WHO [17, 18]. Among 23 African countries surveyed on IPTp coverage levels in 2016, an estimated 19% of eligible pregnant women reported receiving the recommended 3 or more doses of IPTp, compared with 18% in 2015 and 13% in 2014 [19].

While some major progress has been made, the burden of malaria is still high in sub-Saharan Africa where an estimated 30 million pregnant women are at risk of contracting the infection yearly [17, 20]. In one of these countries (Ivory Coast), malaria infection in pregnant women has been reported to be the primary cause of anemia and fetal growth retardation, miscarriages, stillbirth as well as acute illness, pregnancy loss or preterm delivery, and early neonatal mortality [21–23].

With regards to maternal and neonatal tetanus (MNT), it is caused by a potent neurotoxin that is produced by *clostridium tetani*, a common toxic bacterium in soil and in animal intestinal tracts [24]. Tetanus is characterized by painful muscle spasms, serious complications, and can eventually lead to death [25]. Neonatal tetanus (NT) is particularly common and serious in rural areas where most deliveries take place under unhygienic conditions at home where sub-standard prenatal and postnatal care for childbirth prevail. Most of the infected infants do not survive or experience seriously debilitating outcomes. In 2013, WHO estimated about 49,000 neonatal deaths were caused by NT alone [26]. Besides neonates, people of all ages can get tetanus, but it can be prevented by the administration of tetanus toxoid (TT), which induces specific antibodies. To prevent maternal and neonatal tetanus (MNT), TT immunization needs to be given to the mother before or during pregnancy, while also ensuring clean delivery and good cord care practices [27, 28]. Some studies have reported that the uptake of IPTp-SP and TT immunization are associated with women’s level of education, age, residency, wealth index, parity, mass media use and distances from women’s home to health facilities [29, 30]. Also, it has been reported that antenatal care (ANC) visits increases the uptake of IPTp-SP and TT immunization [31–34]. However, in Ivory Coast the proportion of women who take up IPTp-SP and TT while seeking ANC services and the factors associated with this is not known. The objective of this study therefore was to determine the proportion of pregnant women who use IPTp-SP and TT immunization, and the association between women’s uptake of IPTp-SP and TT immunization with antenatal care use in Ivory Coast.

## Methods

### Data source

Data were collected from the fifth round of the Multiple Indicator Cluster Survey (MICS) for Ivory Coast, which was conducted in 2016. The Fifth Multiple Indicator Cluster Survey (MICS 5) in Ivory Coast was initiated by the Ministry of Planning and Development and executed in 2016 by the National Institute of Statistics (INS), as part of the global program for MICS surveys that have been developed by UNICEF since 1990 as an international household survey program. This program aims to support countries in collecting internationally comparable data across a wide range of indicators. Among other things, the MICS 5 Survey provides: (i) recent and detailed information for assessment of the situation of children and women in Ivory Coast; (ii) basic data to evaluate the progress towards the Millennium Development Goals (MDGs), follow the Sustainable Development Goals (SDGs) and National Development Office (PND) 2016–2020. The main objective of the survey was to generate quality data on maternal and child health to facilitate evidence-based policy making, program monitoring towards the Sustainable Development Goals. In this survey 12,463 women were selected from 512 clusters (about 25 households per cluster), of whom 11,780 were interviewed (Response rate 94.5%). Details of the survey have been published elsewhere [35].

### Outcome variables

The outcome variables in this study are similar to the one used in our previous study [30], namely “adequate use of Tetanus toxoid (TT)” and “intermittent preventive therapy with sulfadoxine-pyrimethamine (IPTp-SP)”. These variables were measured by asking the respondents whether or not they received 1) TT vaccination and 2) Fansidar (sulfadoxine-pyrimethamine, SP) during their last pregnancy. According to WHO recommendations, at least three doses of TT (> 2 doses) was defined as adequate and < 2 doses, inadequate. For IPTp-SP, at least three (≥3 doses) doses were defined as adequate while less than three doses (< 3 doses) were considered inadequate.

### Control variables

The following variables were considered for controlling the analysis due to their known /theoretical association with utilization of TT vaccination in the general population:

Age groups (15–19, 20–24, 25–29, 30–34, 35–39, 40–44, 45–49); Residency (Urban, Rural); Region (North, South, West); Education (Pre-Primary/ None, Primary, Lower Secondary, Upper Secondary/higher); Ethnicity (Akan Mandé Du Nord, Gur Ethnic, Non-Ivorian, Other); Wealth status (Poorest, Second, Middle, Fourth, Richest) [36]; Parity (1/2, 3/4, > 4); Radio use (Not At All, Less Than Once A Week, Almost Every Day); TV use (Not At All, Less Than Once A Week, Almost Every Day); Internet use (Yes, No); ANC visit (less than four/ Inadequate, > 4/Adequate).

### Statistical analyses

Data analysis was performed using Stata version 14. Prevalence rates of taking TT vaccination and IPTp-SP for each explanatory variable were shown as percentages by using the survey command to account for survey weights. In the next step, we performed binary logistic regression modes to calculate the odds ratios of the associations between taking TT vaccinations, IPTp-SP and the covariates. Chi-squared bivariate tests and binary logistic regression analyses were used to examine the association. Variables that showed significant association in the bivariate tests were used for the multivariate analysis. For multivariate analyses, separate analyses were performed for rural and urban sample along with the pooled analysis. We did additional tests by including interactions terms such as wealth#education and education#ANC. No significant interactions were observed in the total or stratified (urban /rural) models (results not shown). Statistical significance was set at *p*-value of < 0.05 for all analyses.

## Results

A total of 9583 pregnant women aged 15–49 years were included in this study. Out of this, 81.97% received TT immunization, 78.75%received at least two doses of TT immunization, 17.83% received IPTp-SP drug and 35.46% received at least three doses of IPTp-SP drug. The proportion of TT immunization was 27% among women aged 25–29 years, 69.6% were residing in rural areas (69.6%), 62.7% among women with no formal education, 25.9% among participants from the Akan ethnicity, 27.3% among women from households with the poorest wealth quintile, 43.7% among those who have given birth at least once. Among women who did not use newspaper, radio and TV, the proportion of TT immunization was 71.3, 63.2, and 47.6% respectively. More than half (53.8%) of the women who received TT immunization had had at least three ANC visits.

Higher proportions of women who took at least two doses of TT immunization were observed among women aged 20–24 years (26.5%), women who lived in the rural areas (69.6%), those who had never attended any formal education (60.9%), and participants who belonged to the Akan ethnic group (27.7%). The results of this study also show that the percentage of women who took at least two doses of TT immunization was higher among pregnant women with the poorest households wealth (24.9%), those who had delivered at least once (46.6%), those who did not use newspaper (72.1%), radio (62.1%) nor watched TV almost every day (49.9%), and those who attended at least three ANC visits (60.2%).

In this study, most women using IPTp-SP drugs were in the age group of 25–29 years (28.2%), women who lived in rural areas (69.8%), those who had no formal education (61.7%), participants from the Akan ethnicity (28.7%), those from households with the poorest wealth quintile (27.9%) and women who had delivered at least once (42.4%). It also shows that the proportion of taking IPTp-SP drugs was higher among participants who did not use newspaper (71.1%) and radio (64.2%), watched TV almost every day (47.4%), as well as women who attended at least three ANC visits (53.2%).

Table 1 shows that uptake of three doses of IPTp-SP drugs was higher among participants aged 25–29 years (28.4%), those who lived in rural areas (66.2%), had no formal education (56.3%), women who belonged to the Akan ethnic group (32.7%), women from households with the middle wealth quintile (24.1%), and 45.7% of them had delivered at least once. Also, majority of participants who received three doses of IPTp-SP did not use newspaper, radio and TV respectively (75, 62.6 and 40.4%) as well as those who made at least three ANC visits (70.7%).

**Table 1:** Sample characteristics (*n* = 9583)

	Took TT		Two doses of TT		IPTp-SP		Three doses of IPTP-SP drug	
	Yes (81.97%)	No (18.03%)	Yes (78.75%)	No (21.25%)	Yes (17.83%)	No (82.17%)	Yes (35.46%)	No (64.54%)
Age groups (years)								
15–19	11.8	9.0	12.2	11.8	11.5	21.0	10.7	12.0
20–24	25.0	22.0	26.5	25.0	23.4	17.3	23.5	23.3
25–29	27.0	27.1	26.2	27.0	28.2	16.4	28.4	28.1
30–34	20.5	21.1	20.7	20.5	20.5	15.1	22.0	19.7
35–39	11.2	12.8	10.1	11.2	11.7	12.9	10.9	12.1
40–44	3.9	6.7	3.9	3.9	3.8	10.1	4.1	3.7
45–49	0.6	1.4	0.3	0.6	0.9	7.3	0.5	1.2
p-value	0.002		.003		<.001		.105	
Residency								
Urban	30.4	15.5	30.4	37.5	30.2	42.3	33.8	30.2
Rural	69.6	84.5	69.6	62.5	69.8	57.7	66.2	69.8
p-value	<.001		.103		<.001		.004	
Education								
None	62.7	79.5	60.9	57.0	61.7	54.6	56.3	61.8
Primary	23.1	16.0	24.1	26.3	23.5	21.6	24.4	23.5
Secondary/ Higher	14.2	4.6	15.0	16.7	14.7	23.8	19.3	
p-value	<.001		.021		.170		<.001	
Ethnicity								
Other	12.6	7.6	12.2	12.6	12.6	12.4	12.1	12.6
Akan	25.9	16.7	27.7	25.9	28.7	29.8	32.7	28.7
Mandé Du Nord	16.8	15.4	16.8	16.8	13.2	17.5	11.9	13.2
Gur	19.5	36.5	18.9	19.5	21.1	19.1	20.9	21.1
Non-Ivorian	25.2	23.9	24.3	25.1	24.5	21.2	22.3	24.5
p-value	<.001		.046		<.001		.026	
Wealth index								
Poorest	27.3	46.0	24.9	27.3	27.9	19.7	23.6	27.9
Poorer	24.5	28.1	24.2	24.5	25.0	19.5	22.0	25.0
Middle	22.6	15.4	23.7	22.6	22.3	19.6	24.1	22.3
Richer	15.3	6.7	17.0	15.3	15.1	17.2	18.7	15.1
Richest	10.3	3.8	10.1	10.4	9.8	24.0	11.7	9.8
p-value	<.001		.001		<.001		<.001	
Parity								
1/2	43.7	12.2	46.6	11.8	42.4	26.8	45.7	41.9
3/4	30.5	13.3	29.4	17.0	30.8	12.3	28.4	30.7
> 4	25.8	74.4	23.9	71.3	26.7	60.9	25.9	27.4
p-value	<.001		<.001		<.001		.060	
Newspaper								
Almost Everyday	11.8	16.3	11.3	25.5	12.3	16.3	10.6	12.3
Few days a week	17.0	8.1	16.6	11.4	16.6	20.2	14.4	16.6
Never	71.3	75.7	72.1	63.2	71.1	63.5	75.0	71.1
p-value	.159		.114		.050		.105	
Radio								
Almost Everyday	25.5	26.9	26.0	25.5	24.5	27.4	26.0	24.5
Few days a week	11.4	5.9	11.9	11.4	11.3	12.6	11.4	11.3
Never	63.2	67.2	62.1	63.2	64.2	60.1	62.6	64.2
p-value	<.001		.166		.133		.137	
TV								
Almost Everyday	47.6	43.9	49.9	47.6	47.4	55.1	51.1	47.4
Few days a week	7.7	7.4	7.5	7.7	7.5	7.3	8.5	7.5
Never	44.7	48.7	42.6	44.7	45.1	37.5	40.4	45.1
p-value	<.001		.004		.047		.003	
Number of ANC visits								
< 3	46.2	96.7	39.8	46.2	46.8	49.0	29.3	46.8
3/3+	53.8	3.4	60.2	53.8	53.2	51.0	70.7	53.2
*p*-value	<.001		<.001		<.001		<.001	

Table 2 shows the results of multivariable regression model. The findings of this study showed that women’s age, ethnicity and number of ANC visits were significantly associated with taking adequate doses of IPTp-SP drugs. Whereas, women’s educational level and number of ANC visits were statistically associated with increased odds of taking IPTp-SP drugs. Women of age group 25–29 years had higher odds (OR = 2.028, 95%CI = 1.120–3.669) of taking the adequate doses of IPTp-SP drugs compared to those aged 15–19 years. When stratified by areas, this positive association was observed only for urban women aged 25–29 years (OR = 3.943, 95%CI = 1.431–10.86) compared to those aged 15–19 years. Having primary and secondary/high educational level increased the odds of taking IPTp-SP drugs (OR = 2.504, 95%CI = 1.020–6.146) and (3.298,95%CI = 1.343–8.097) respectively, compared to those with no formal education. Stratified by areas, the significant association between education and IPTp-SP drugs was true for urban women only who had secondary/high educational level (OR = 3.212,95%CI = 1.002–10.29) compared to those with no formal education. Similarly, we found a strong association between ANC visits and IPTp-SP drugs (OR = 1.656, 95%CI = 1.194–2.299). Upon stratification by areas, the odds of taking IPTp-SP drugs were significantly higher among women who attended at least 4 ANC visits in both urban (OR = 2.126, 95%CI = 1.214–3.724) and rural areas (OR = 1.597, 95%CI = 1.037–2.459) compared to those who attended less than 4 ANC visits. Our results also indicated that IPTp-SP doses was associated with 4 ANC visits (OR = 3.291, 95%CI = 2.157–5.020) compared to counterparts who attended less than 4 ANC visits. Also, the probability of taking adequate doses of IPTp-SP was higher among women with at least 4 ANC visits in both urban (OR = 2.777, 95%CI = 1.286–5.999) and rural areas (OR = 4.087, 95%CI = 2.350–7.107) compared to those who had less than 4 ANC visits. However, the odds of taking the adequate doses of IPTp-SP drugs were lower among women who were from Mandé Du Nord ethnic group (OR = 0.378,95%CI = 0.145–0.983) compared to those from another ethnicity.

**Table 2:** Correlates of using IPTp-SP drugs among pregnant women in Ivory Coast

	Antimalarial drug			Adequate dose of antimalarial drug		
	Total	Urban (*n* = 3845)	Rural (*n* = 5738)	Total	Urban (*n* = 3845)	Rural (*n* = 5738)
Age groups (15–19)						
20–24	0.779	0.580	0.954	1.665	1.803	1.662
	[0.482,1.258]	[0.256,1.313]	[0.511,1.783]	[0.939,2.955]	[0.637,5.107]	[0.781,3.539]
25–29	1.410	1.327	1.476	2.028^*^	3.943^**^	1.479
	[0.832,2.388]	[0.575,3.061]	[0.715,3.049]	[1.120,3.669]	[1.431,10.86]	[0.653,3.347]
30–34	1.166	1.060	1.135	1.945	2.860	1.975
	[0.642,2.118]	[0.429,2.616]	[0.475,2.714]	[0.977,3.872]	[0.965,8.480]	[0.701,5.567]
35–39	1.821	2.215	1.576	2.197	2.130	2.335
	[0.841,3.941]	[0.690,7.109]	[0.500,4.969]	[0.935,5.162]	[0.577,7.863]	[0.622,8.763]
40–49	1.200	3.624	0.651	1.750	1.807	2.179
	[0.408,3.523]	[0.513,25.59]	[0.156,2.708]	[0.535,5.719]	[0.276,11.84]	[0.383,12.39]
Residency (Urban)						
Rural	1.010	NA	NA	1.314	NA	NA
	[0.654,1.559]			[0.815,2.118]		
Education (None)						
Primary	2.504^*^	2.883	2.810	1.276	2.446	0.790
	[1.020,6.146]	[0.872,9.532]	[0.596,13.24]	[0.345,4.710]	[0.380,15.74]	[0.0950,6.574]
Secondary/higher	3.298^**^	3.212^*^	4.309	1.667	1.928	1.565
	[1.343,8.097]	[1.002,10.29]	[0.889,20.88]	[0.452,6.153]	[0.306,12.15]	[0.184,13.32]
Ethnicity (Other)						
Akan	1.051	0.875	1.214	0.784	0.500	0.909
	[0.671,1.645]	[0.441,1.735]	[0.630,2.341]	[0.464,1.325]	[0.220,1.137]	[0.406,2.031]
Mandé Du Nord	0.927	0.543	2.762	0.702	0.378^*^	0.754
	[0.519,1.656]	[0.250,1.178]	[0.876,8.711]	[0.349,1.411]	[0.145,0.983]	[0.178,3.187]
Gur	0.797	0.653	1.111	0.542	0.392	0.364
	[0.442,1.438]	[0.277,1.537]	[0.450,2.741]	[0.272,1.080]	[0.148,1.038]	[0.111,1.201]
Non-Ivorian	0.829	0.811	0.839	0.554	0.405	0.489
	[0.496,1.385]	[0.367,1.795]	[0.406,1.732]	[0.293,1.044]	[0.153,1.071]	[0.181,1.322]
Wealth (Poorest)						
Poorer	0.770	1.169	0.685	1.149	1.114	0.960
	[0.456,1.300]	[0.657,2.521]	[0.393,1.196]	[0.614,2.153]	[0.551,2.121]	[0.485,1.902]
Middle	0.884	0.690	0.794	1.336	0.337	1.239
	[0.499,1.567]	[0.143,3.326]	[0.414,1.523]	[0.690,2.587]	[0.0725,1.567]	[0.563,2.727]
Higher	0.663	0.406	0.659	1.264	0.460	1.084
	[0.340,1.293]	[0.0853,1.933]	[0.286,1.514]	[0.579,2.760]	[0.0985,2.148]	[0.397,2.955]
Highest	0.689	0.399	1.229	0.962	0.458	0.522
	[0.331,1.434]	[0.0799,1.990]	[0.383,3.942]	[0.414,2.238]	[0.0950,2.206]	[0.135,2.022]
Newspaper (Never)						
Few days a week	1.047	1.632	0.512	1.025	1.024	1.002
	[0.601,1.825]	[0.806,3.304]	[0.182,1.441]	[0.542,1.938]	[0.455,2.308]	[0.310,3.242]
Almost everyday	1.211	1.467	0.848	1.575	1.380	1.901
	[0.746,1.965]	[0.788,2.731]	[0.348,2.067]	[0.907,2.734]	[0.663,2.873]	[0.723,4.999]
Radio (Never)						
Few days a week	1.129	1.046	1.207	1.263	0.676	2.129
	[0.733,1.738]	[0.549,1.993]	[0.648,2.247]	[0.764,2.086]	[0.320,1.429]	[0.973,4.661]
Almost everyday	1.188	0.850	1.460	1.104	0.917	1.436
	[0.846,1.668]	[0.508,1.422]	[0.901,2.363]	[0.747,1.633]	[0.506,1.660]	[0.791,2.608]
TV (Never)						
Few days a week	0.813	1.616	0.736	1.084	0.371	1.292
	[0.455,1.455]	[0.451,5.788]	[0.364,1.490]	[0.552,2.127]	[0.0682,2.014]	[0.540,3.092]
Almost everyday	0.658	0.982	0.592	1.102	1.820	1.078
	[0.423,1.026]	[0.377,2.558]	[0.346,1.013]	[0.662,1.835]	[0.624,5.308]	[0.560,2.074]
Parity (1/2)						
3/4	0.954	0.931	0.994	0.819	1.226	0.628
	[0.637,1.427]	[0.511,1.697]	[0.550,1.797]	[0.518,1.295]	[0.616,2.441]	[0.315,1.255]
> 4	0.795	0.489	1.069	0.921	1.177	0.676
	[0.432,1.462]	[0.173,1.376]	[0.457,2.500]	[0.458,1.851]	[0.360,3.846]	[0.257,1.780]
ANC (< 4)						
4	1.656^**^	2.126^**^	1.597^*^	3.291^***^	2.777^**^	4.087^***^
	[1.194,2.299]	[1.214,3.724]	[1.037,2.459]	[2.157,5.020]	[1.286,5.999]	[2.350,7.107]

Exponentiated coefficients; 95% confidence intervals in brackets

^*^ *p* < 0.05, ^**^ *p* < 0.01, ^***^ *p* < 0.001

Table 3 shows the correlates of using TT immunization among pregnant women in Ivory Coast. After adjusting for possible confounding by each of the covariates in the multivariate logistic regression, pregnant women’s age, parity and number of ANC visits were associated with TT immunization. Only ANC visits was significantly associated with a higher odd of receiving the adequate doses of TT immunization.

**Table 3:** Correlates of using tetanus toxoid among pregnant women in Ivory Coast

	Tetanus toxoid			Adequate dose of tetanus toxoid		
	Total	Urban (*n* = 3845)	Rural (*n* = 5738)	Total	Urban (*n* = 3845)	Rural (*n* = 5738)
Age groups (15–19)						
20–24	1.168	1.320	1.353	1.147	1.509	1.126
	[0.494,2.759]	[0.183,9.516]	[0.492,3.722]	[0.692,1.903]	[0.661,3.447]	[0.576,2.201]
25–29	1.249	0.463	3.061	0.831	1.526	0.579
	[0.497,3.143]	[0.0821,2.609]	[0.825,11.36]	[0.489,1.411]	[0.665,3.500]	[0.278,1.205]
30–34	0.867	0.337	2.574	0.654	0.952	0.568
	[0.311,2.417]	[0.0535,2.120]	[0.545,12.16]	[0.355,1.208]	[0.390,2.325]	[0.229,1.407]
35–39	1.386	0.449	7.842^*^	0.621	1.077	0.435
	[0.393,4.887]	[0.0556,3.629]	[1.088,56.50]	[0.289,1.335]	[0.343,3.382]	[0.141,1.341]
40–49	0.858	07942	1.002	0.450	0.316	0.499
	[0.184,3.991]	[0.490,2.012]	[0.144,6.969]	[0.156,1.301]	[0.0583,1.715]	[0.112,2.223]
Residency (Urban)						
Rural	0.803	NA	NA	0.676	NA	NA
	[0.388,1.662]			[0.433,1.055]		
Education (None)						
Primary	0.913	1.117	1.653	1.042	1.065	0.830
	[0.189,4.410]	[0.101,12.31]	[0.145,18.85]	[0.389,2.790]	[0.276,4.117]	[0.164,4.195]
Secondary/higher	1.259	1.476	2.951	1.013	0.800	0.958
	[0.257,6.164]	[0.139,15.69]	[0.239,36.51]	[0.379,2.710]	[0.215,2.973]	[0.185,4.951]
Ethnicity (Other)						
Akan	0.841	0.553	1.720	1.050	1.076	1.108
	[0.367,1.929]	[0.126,2.432]	[0.560,5.284]	[0.667,1.651]	[0.533,2.171]	[0.584,2.104]
Mandé Du Nord	0.465	0.414	0.325	1.412	1.551	1.876
	[0.169,1.278]	[0.0863,1.984]	[0.0610,1.732]	[0.775,2.574]	[0.701,3.432]	[0.636,5.529]
Gur	0.883	0.415	5.587	0.589	0.583	0.457
	[0.310,2.517]	[0.0821,2.103]	[0.914,34.15]	[0.325,1.068]	[0.245,1.390]	[0.181,1.151]
Non-Ivorian	0.663	1.442	0.476	0.902	0.993	0.831
	[0.258,1.701]	[0.201,10.33]	[0.147,1.542]	[0.533,1.528]	[0.443,2.225]	[0.396,1.746]
Wealth (Poorest)						
Poorer	1.294	1.219	1.252	0.899	0.813	0.914
	[0.566,2.958]	[0.663,2.381]	[0.513,3.058]	[0.524,1.543]	[0.589,2.610]	[0.516,1.617]
Middle	1.550	2.934	1.453	1.237	2.283	1.285
	[0.614,3.915]	[0.167,51.57]	[0.471,4.483]	[0.696,2.199]	[0.498,10.47]	[0.664,2.488]
Higher	0.824	2.485	0.539	1.190	2.214	1.073
	[0.289,2.348]	[0.144,42.82]	[0.140,2.068]	[0.603,2.349]	[0.485,10.11]	[0.464,2.481]
Highest	1.148	4.839	0.353	0.774	1.427	0.859
	[0.336,3.919]	[0.249,94.02]	[0.0512,2.438]	[0.370,1.620]	[0.302,6.738]	[0.282,2.618]
Newspaper (Never)						
Few days a week	1.516	2.328	0.853	1.096	1.050	0.976
	[0.618,3.719]	[0.703,7.709]	[0.161,4.526]	[0.628,1.912]	[0.523,2.107]	[0.350,2.726]
Almost everyday	1.794	2.315	0.938	1.284	1.431	0.837
	[0.829,3.884]	[0.793,6.759]	[0.233,3.767]	[0.789,2.090]	[0.759,2.698]	[0.351,1.997]
Radio (Never)						
Few days a week	0.846	1.488	0.512	1.270	1.886	0.885
	[0.409,1.750]	[0.421,5.268]	[0.188,1.391]	[0.807,1.999]	[0.944,3.769]	[0.463,1.693]
Almost everyday	0.896	0.735	1.068	1.014	1.082	1.025
	[0.499,1.608]	[0.291,1.856]	[0.455,2.506]	[0.719,1.430]	[0.648,1.808]	[0.629,1.670]
Radio (Never)						
Few days a week	0.723	0.992	0.328	0.854	0.659	1.088
	[0.298,1.756]	[0.0807,12.20]	[0.107,1.003]	[0.473,1.543]	[0.199,2.185]	[0.522,2.267]
Almost everyday	0.951	0.513	0.463	1.266	1.183	1.420
	[0.448,2.018]	[0.489,1.417]	[0.177,1.216]	[0.808,1.982]	[0.458,3.056]	[0.829,2.432]
Parity (1/2)						
3/4	0.615	0.445	1.050	1.341	1.435	1.301
	[0.308,1.227]	[0.166,1.197]	[0.362,3.044]	[0.888,2.025]	[0.774,2.663]	[0.715,2.369]
> 4	0.449	0.694	0.218^*^	1.400	1.886	1.243
	[0.175,1.149]	[0.134,3.597]	[0.0552,0.858]	[0.756,2.594]	[0.647,5.497]	[0.536,2.880]
ANC (< 4)						
4	2.347^**^	2.493	2.397^*^	1.968^***^	1.531	2.387^***^
	[1.384,3.981]	[0.982,6.331]	[1.172,4.901]	[1.398,2.771]	[0.835,2.807]	[1.529,3.726]

Exponentiated coefficients; 95% confidence intervals in brackets

^*^ *p* < 0.05, ^**^ *p* < 0.01, ^***^ *p* < 0.001

Accordingly, the probability of using TT immunization was significantly higher among women aged 35–39 years (OR = 7.842, 95%CI = 1.088–56.50) compared to those 15–19 years old. This result is true for rural women only. Having higher parity (> 4) was significantly associated with lower odds of receiving TT immunization for rural participants only (OR = 0.218, 95%CI = 0.055–0.858) compared to those who had delivered at least once. The model also reveals that women who had attended 4 ANC visits markedly increased the odds of receiving TT immunization (OR = 2.347, 95%CI = 1.384–3.981) compared to those who had attended less. This association was also true for rural women only (OR = 2.397, 95%CI = 1.172–4.901). The odds of receiving the adequate doses of TT immunization were higher among women who had attended 4 ANC visits (OR = 1.968, 95%CI = 1.398–2.771) compared to those with less ANC attendance. This observation is similar for women in rural areas (OR = 2.387, 95%CI = 1.529–3.726), but different for women in urban areas.

## Discussion

Despite numerous government interventions, tetanus and malaria related morbidity and mortalities remain a significant public health challenge across Africa. In the last few years, public health programs have been trying to promote preventive techniques such as the use of tetanus toxoid and IPTp-SP, however, challenges remain in increasing the prevalence of these highly cost-effective strategies.

In Ivory Coast, the current policies for malaria and TT immunization are those recommended by WHO for the prevention of malaria and tetanus during pregnancy using intermittent preventive treatment with sulfadoxine pyrimethamine and TT vaccination respectively. These policies are excellent with a high potential of reducing the burden of these diseases in this country. However, implementation of these policies is limited, and women’s uptake of the vaccines sometimes hindered by partners objecting to it [37, 38].

In this study, we found the proportion of women who took IPTp-SP drugs (17.83%) and in adequate doses (35.46%) was much lower than that of those who received TT immunization and in adequate doses among pregnant Ivory Coast women. This observation is dissimilar to the previous studies that found higher rates of taking IPTp-SP drugs [18, 39, 40]. This disparity could be influenced by distance of health facilities from the women’s homes, low education of the women, household income level of the women and lack of information about the effectiveness of taking IPTp-SP drugs.

Using regression analysis model, we found that women’s age was an important indicator of the taking of both IPTp-SP drugs and TT immunization. For instance, women aged 25–29 years had higher odds of receiving adequate doses of IPTp-SP drugs compared to those aged 15–19 years. This finding is in line with previous cross-sectional studies conducted in Malawi and Tanzania [32, 41]. Similarly, the odds of TT immunization usage were significantly higher among women aged 35–39 years compared to those aged 15–19 years. However, the association was significant for only rural women. This finding is consistent with the study by Dubale and colleagues, that involved women in Ethiopia also showing an association between higher age and utilization of TT immunization [42]. There is a possibility that women in higher age groups have been exposed to multiple pregnancies and also had opportunity to attend more ANC visits where information about TT immunization was given compared to their younger counterparts.

We also found that participants who attended at least four ANC visits had higher odds of taking IPTp-SP drugs and TT immunization compared to those who attended less. Having at least four ANC visits was significantly associated with higher odds of receiving adequate doses of IPTp-SP drugs and TT immunization compared to those who attended less than four ANC visits. The probability of taking IPTp-SP drugs was significantly higher among pregnant women who had at least four ANC visits during the last pregnancy compared to their counterparts. This finding with regard to healthcare service is similar to studies conducted in Ghana [43, 44], Malawi [32, 45], Mali [46], Cameroon [47], and Tanzania [48, 49]. Concerning adequate doses of TT immunization, our findings also revealed that women who attended at least four ANC visits were more likely to have been immunized with protective doses of TT compared to those who made fewer visits. Other studies also verified this association [33, 50, 51]. This is perhaps because women who attend more ANC visits are more likely to be informed about the importance of IPTp-SP drugs and would therefore receive it than those who attended less.

There was a significant association between utilization of TT immunization and participant’s parity. Our study revealed that women with higher parity had lower odds of TT immunization usage compared to those of lower parity (1–2). This was supported by findings from previous studies that have found higher parity (> 4 pregnancies) to be a significant predictor of TT immunization [52, 53]. The possible reason may be due to women’s previous experience with delivery and the side effects of TT immunization. It’s also possible that women with higher parity are less likely to have better education and employment. This hypothesis is supported by the present findings as well. For instance, women with primary and secondary/higher educational level had higher odds of taking IPTp-SP drugs compared to those with no education. This result is consistent with previous studies done in Malawi, Uganda and Ghana in which high maternal education was significantly associated with high uptake of IPTp-SP drugs [18, 54–57]. This is likely because education can empower women to take better and more effective decisions regarding their health issues, which can contribute to adequate utilization of healthcare services like IPTp-SP drugs including ANC attendance. Women of Mendé ethnicity had lower odds of receiving adequate doses of IPTp-SP drugs compared to those from other ethnicities. There was no evidence in Africa, but ethnic differences were found in US [58–61]. Possible explanation could be to the lack of confidence in vaccination among ethnic groups [62, 63].

### Strengths and limitations

The current study was not without limitations. Firstly, the study was based on secondary data from a cross sectional survey. Hence, it cannot be used to infer causality but only to show association between variables [64, 65]. Secondly, the data was collected three years ago, and although the disparity in taking IPTp-SP drugs and TT immunization still exists, the underlying causes may have changed. As strengths, the sample size was large, and the findings can be generalized for women aged 15–49 years. The cross-sectional nature of the data prevented from making any causal assumptions. The prevalence of IPT-SP and TT use was self-reported and therefore remains subject to reporting bias. We were also unable to assess during which trimester the tetanus toxoid and IPTp-SP were administered. Future studies should be conducted aiming at establishing the link of causality between ANC attendance and TT vaccination/IPTp-SP coverage.

## Conclusion

In general, our results indicate that uptake of these vaccines is low with adequate IPTp-SP dose uptake much lower than adequate TT dose uptake. Age, education level, parity, ethnicity and number of ANC visits were found to be significantly associated with taking IPTp-SP drugs and TT immunization and their adequate doses respectively. Vaccination promotion efforts need to be intensified to protect pregnant women and could reduce adverse health outcomes among the newborn in Ivory Coast.

## Data Availability

Data for this study are available in the public domain of the UNICEF website: https://mics-surveys-prod.s3.amazonaws.com/MICS5/West%20and%20Central%20Africa/Côte%20d%27Ivoire/2016/Final/Cote%20d%27Ivoire%202016%20MICS_French.pdf

## Abbreviations

ANC: Antenatal care
CI: Confidence interval
IPTp-SP: Intermittent preventive treatment of malaria during pregnancy with sulfadoxine-pyrimethamine
MICS: Multiple Indicator Cluster Survey
OR: Odds ratio
TT: Tetanus toxoid
WHO: World Health Organization

## References

[1] Desai M, ter Kuile FO, Nosten F, McGready R, Asamoa K, Brabin B (2007). Epidemiology and burden of malaria in pregnancy. Lancet Infect Dis.

[2] Stanfield JP, Galazka A (1984). Neonatal tetanus in the world today. Bull World Health Organ.

[3] World Health Organization. Electronic address: sageexecsec@who.int. Tetanus vaccines: WHO position paper, February 2017 - Recommendations. Vaccine. 2018; 36:3573–5. 10.1016/j.vaccine.2017.02.034 28427847

[4] Yaya S, Uthman OA, Amouzou A, Bishwajit G (2018). Use of intermittent preventive treatment among pregnant women in sub-Saharan Africa: evidence from malaria Indicator surveys. Tropical Medicine and Infectious Disease.

[5] WHO. Fact sheet about Malaria. 2019. https://www.who.int/news-room/fact-sheets/detail/malaria. .

[6] Antinori Spinello, Galimberti Laura, Milazzo Laura, Corbellino Mario (2012). BIOLOGY OF HUMAN MALARIA PLASMODIA INCLUDING PLASMODIUM KNOWLESI. Mediterranean Journal of Hematology and Infectious Diseases.

[7] WHO. World malaria report. Switzerland: Geneva; 2018.

[8] Onyeneho NG (2013). Sleeping under insecticide-treated nets to prevent malaria in Nigeria: what do we know?. J Health Popul Nutr.

[9] Radeva-Petrova D, Kayentao K, ter Kuile FO, Sinclair D, Garner P. Drugs for preventing malaria in pregnant women in endemic areas: any drug regimen versus placebo or no treatment Cochrane Database of Systematic Reviews. 2014. 10.1002/14651858.CD000169.pub3. 10.1002/14651858.CD000169.pub3 PMC4498495 25300703

[10] Steinhardt LC, Jean YS, Impoinvil D, Mace KE, Wiegand R, Huber CS (2017). Effectiveness of insecticide-treated bednets in malaria prevention in Haiti: a case-control study. Lancet Glob Health.

[11] Dengela D, Seyoum A, Lucas B, Johns B, George K, Belemvire A (2018). Multi-country assessment of residual bio-efficacy of insecticides used for indoor residual spraying in malaria control on different surface types: results from program monitoring in 17 PMI/USAID-supported IRS countries. Parasit Vectors.

[12] Kigozi R, Baxi SM, Gasasira A, Sserwanga A, Kakeeto S, Nasr S (2012). Indoor residual spraying of insecticide and malaria morbidity in a high transmission intensity area of Uganda. PLoS One.

[13] Steinhardt LC, Yeka A, Nasr S, Wiegand RE, Rubahika D, Sserwanga A (2013). The effect of indoor residual spraying on malaria and Anemia in a high-transmission area of northern Uganda. Am J Trop Med Hyg..

[14] Keiser J, Singer BH, Utzinger J (2005). Reducing the burden of malaria in different eco-epidemiological settings with environmental management: a systematic review. Lancet Infect Dis.

[15] Mokuolu OA, Coker AO, Adejumo M, Sridhar MKC (2018). Modeling a covered drainage system for the reduction of malaria prevalence. Ain Shams Engineering Journal.

[16] Randell HF, Dickinson KL, Shayo EH, Mboera LEG, Kramer RA (2010). Environmental management for malaria control: knowledge and practices in Mvomero. Tanzania Ecohealth.

[17] Henry M, Florey L, Youll S, Gutman JR (2018). An analysis of country adoption and implementation of the 2012 WHO recommendations for intermittent preventive treatment for pregnant women in sub-Saharan Africa. Malar J.

[18] Orish VN, Onyeabor OS, Boampong JN, Afoakwah R, Nwaefuna E, Acquah S (2015). Prevalence of intermittent preventive treatment with sulphadoxine-pyrimethamine (IPTp-SP) use during pregnancy and other associated factors in Sekondi-Takoradi. Ghana Afr Health Sci.

[19] WHO (World Health Organization). World malaria report. Switzerland: Geneva; 2017. https://www.who.int/malaria/media/world-malaria-report-2017/en/.

[20] Steketee RW, Nahlen BL, Parise ME, Menendez C (2001). The burden of malaria in pregnancy in malaria-endemic areas. Am J Trop Med Hyg.

[21] van Eijk AM, Hill J, Noor AM, Snow RW, ter Kuile FO (2015). Prevalence of malaria infection in pregnant women compared with children for tracking malaria transmission in sub-Saharan Africa: a systematic review and meta-analysis. Lancet Glob Health.

[22] Koné S, Hürlimann E, Baikoro N, Dao D, Bonfoh B, N’Goran EK (2018). Pregnancy-related morbidity and risk factors for fatal foetal outcomes in the Taabo health and demographic surveillance system. Côte d’Ivoire BMC Pregnancy and Childbirth.

[23] Wang H, Bhutta ZA, Coates MM, Coggeshall M, Dandona L, Diallo K (2016). Global, regional, national, and selected subnational levels of stillbirths, neonatal, infant, and under-5 mortality, 1980–2015: a systematic analysis for the global burden of disease study 2015. Lancet.

[24] Roper MH, Vandelaer JH, Gasse FL (2007). Maternal and neonatal tetanus. Lancet..

[25] Aziz R, Colombe S, Mwakisambwe G, Ndezi S, Todd J, Kalluvya S (2018). Pre-post effects of a tetanus care protocol implementation in a sub-Saharan African intensive care unit. PLoS Negl Trop Dis.

[26] Liu L, Oza S, Hogan D, Perin J, Rudan I, Lawn JE (2015). Global, regional, and national causes of child mortality in 2000–13, with projections to inform post-2015 priorities: an updated systematic analysis. Lancet.

[27] Khan R, Vandelaer J, Yakubu A, Raza AA, Zulu F (2015). Maternal and neonatal tetanus elimination: from protecting women and newborns to protecting all. Int J Women's Health.

[28] Thwaites CL, Beeching NJ, Newton CR (2015). Maternal and neonatal tetanus. Lancet.

[29] Anatea MD, Mekonnen TH, Dachew BA. Determinants and perceptions of the utilization of tetanus toxoid immunization among reproductive-age women in Dukem town, eastern Ethiopia: a community-based cross-sectional study. BMC Int Health Hum Rights. 2018;18. 10.1186/s12914-018-0168-0. 10.1186/s12914-018-0168-0 PMC6022706 29950171

[30] Buh Amos, Kota Komlan, Bishwajit Ghose, Yaya Sanni (2019). Prevalence and Associated Factors of Taking Intermittent Preventive Treatment in Pregnancy in Sierra Leone. Tropical Medicine and Infectious Disease.

[31] Abir T, Ogbo FA, Stevens GJ, Page AN, Milton AH, Agho KE (2017). The impact of antenatal care, iron–folic acid supplementation and tetanus toxoid vaccination during pregnancy on child mortality in Bangladesh. PLoS One.

[32] Azizi SC, Chongwe G, Chipukuma H, Jacobs C, Zgambo J, Michelo C. Uptake of intermittent preventive treatment for malaria during pregnancy with Sulphadoxine-Pyrimethamine (IPTp-SP) among postpartum women in Zomba District, Malawi: a cross-sectional study. BMC Pregnancy Childbirth. 2018;18. 10.1186/s12884-018-1744-y. 10.1186/s12884-018-1744-y PMC5910602 29678150

[33] Mihret MS, Limenih MA, Gudayu TW. The role of timely initiation of antenatal care on protective dose tetanus toxoid immunization: the case of northern Ethiopia post natal mothers. BMC Pregnancy Childbirth. 2018;18. 10.1186/s12884-018-1878-y. 10.1186/s12884-018-1878-y PMC6003212 29907139

[34] Toure OA, Kone PL, Coulibaly MA, Ako BA, Gbessi EA, Coulibaly B (2014). Coverage and efficacy of intermittent preventive treatment with sulphadoxine pyrimethamine against malaria in pregnancy in Côte d’Ivoire five years after its implementation. Parasit Vectors.

[35] INS. Institut National de la Statistique: Enquête par grappes à indicateurs multiples, 2016. Rapport des résultats clés 2017.

[36] Bishwajit G (2017). Household wealth status and overweight and obesity among adult women in Bangladesh and Nepal. Obes Sci Pract.

[37] Larson Williams A, Mitrovich R, Mwananyanda L, Gill C (2018). Maternal vaccine knowledge in low- and middle-income countries—and why it matters. Hum Vaccin Immunother..

[38] Yavo J-C, Amari ASG, Assi S-B, Assemian A, Kouamé R, Balayssac E (2019). Evaluation of the knowledge of intermittent preventive treatment during pregnancy (IPTp) with sulfadoxine-pyrimethamine in Ivory Coast. Therapie..

[39] Ibrahim H, Maya ET, Issah K, Apanga PA, Bachan EG, Noora CL. Factors influencing uptake of intermittent preventive treatment of malaria in pregnancy using sulphadoxine pyrimethamine in Sunyani Municipality, Ghana. Pan African Medical Journal. 2017;28. 10.11604/pamj.2017.28.122.12611. 10.11604/pamj.2017.28.122.12611 PMC5839217 29515740

[40] Protas J, Tarimo D, Moshiro C. Determinants of timely uptake of ITN and SP (IPT) and pregnancy time protected against malaria in Bukoba. Tanzania BMC Res Notes. 2016;9. 10.1186/s13104-016-2122-3. 10.1186/s13104-016-2122-3 PMC4915182 27328717

[41] Kibusi SM, Kimunai E, Hines CS. Predictors for uptake of intermittent preventive treatment of malaria in pregnancy (IPTp) in Tanzania. BMC Public Health. 2015;15. 10.1186/s12889-015-1905-0. 10.1186/s12889-015-1905-0 PMC4458339 26049737

[42] Dubale Mamoro Muluken, Kelbiso Hanfore Lolemo (2018). Tetanus Toxoid Immunization Status and Associated Factors among Mothers in Damboya Woreda, Kembata Tembaro Zone, SNNP, Ethiopia. Journal of Nutrition and Metabolism.

[43] Hommerich L, von Oertzen C, Bedu-Addo G, Holmberg V, Acquah PA (2007). Eggelte TA, et al. Owusu Malar J.

[44] Owusu-Boateng I, Anto F (2017). Intermittent preventive treatment of malaria in pregnancy: a cross-sectional survey to assess uptake of the new sulfadoxine–pyrimethamine five dose policy in Ghana. Malar J.

[45] Nkoka O, Chuang T-W, Chen Y-H. Association between timing and number of antenatal care visits on uptake of intermittent preventive treatment for malaria during pregnancy among Malawian women. Malar J. 2018;17. 10.1186/s12936-018-2360-z. 10.1186/s12936-018-2360-z PMC5968590 29793482

[46] Hill Jenny, Kayentao Kassoum, Touré Mahamoudou, Diarwara Sory, Bruce Jane, Smedley James, Doumbo Ogobara K., Kuile Feiko O. ter., Webster Jayne (2014). Effectiveness of Antenatal Clinics to Deliver Intermittent Preventive Treatment and Insecticide Treated Nets for the Control of Malaria in Pregnancy in Mali: A Household Survey. PLoS ONE.

[47] Leonard N, Eric FB, Judith A-KK, Samuel W. Factors associated to the use of insecticide treated nets and intermittent preventive treatment for malaria control during pregnancy in Cameroon. Arch Public Health. 2016;74. 10.1186/s13690-016-0116-1. 10.1186/s13690-016-0116-1 PMC4734861 26835009

[48] Exavery A, Mbaruku G, Mbuyita S, Makemba A, Kinyonge IP, Kweka H (2014). Factors affecting uptake of optimal doses of sulphadoxine-pyrimethamine for intermittent preventive treatment of malaria in pregnancy in six districts of Tanzania. Malar J.

[49] Mpogoro FJ, Matovelo D, Dosani A, Ngallaba S, Mugono M, Mazigo HD (2014). Uptake of intermittent preventive treatment with sulphadoxine-pyrimethamine for malaria during pregnancy and pregnancy outcomes: a cross-sectional study in Geita district. North-Western Tanzania Malar J.

[50] Haile ZT, Chertok IRA, Teweldeberhan AK (2013). Determinants of utilization of sufficient tetanus toxoid immunization during pregnancy: evidence from the Kenya demographic and health survey, 2008–2009. J Community Health.

[51] Naeem M, Khan MZ-I, Abbas SH, Adil M, Khan A, Naz SM (2010). Coverage and factors associated with tetanus toxoid vaccination among married women of reproductive age: a cross sectional study in Peshawar. J Ayub Med Coll Abbottabad.

[52] Maral I, Baykan Z, Aksakal F, Kayikcioglu F, Bumin M (2001). Tetanus immunization in pregnant women: evaluation of maternal tetanus vaccination status and factors affecting rate of vaccination coverage. Public Health.

[53] Pathirana J, Nkambule J, Black S (2015). Determinants of maternal immunization in developing countries. Vaccine..

[54] Addai-Mensah Otchere, Annani-Akollor Max Efui, Fondjo Linda Ahenkorah, Sarbeng Kwadwo, Anto Enoch Odame, Owiredu Eddie-Williams, Arthur Shanice Nglokie (2018). Regular Antenatal Attendance and Education Influence the Uptake of Intermittent Preventive Treatment of Malaria in Pregnancy: A Cross-Sectional Study at the University Hospital, Kumasi, Ghana. Journal of Tropical Medicine.

[55] Holtz TH, Kachur SP, Roberts JM, Marum LH, Mkandala C, Chizani N (2004). Use of antenatal care services and intermittent preventive treatment for malaria among pregnant women in Blantyre District. Malawi Trop Med Int Health.

[56] Sangaré LR, Stergachis A, Brentlinger PE, Richardson BA, Staedke SG, Kiwuwa MS (2010). Determinants of use of intermittent preventive treatment of malaria in pregnancy: Jinja. Uganda PLOS ONE.

[57] Wilson NO, Ceesay FK, Obed SA, Adjei AA, Gyasi RK, Rodney P (2011). Intermittent preventive treatment with Sulfadoxine-Pyrimethamine against malaria and Anemia in pregnant women. Am J Trop Med Hyg..

[58] Egede LE, Zheng D (2003). Racial/ethnic differences in influenza vaccination coverage in high-risk adults. Am J Public Health.

[59] Garza MA, Quinn SC, Li Y, Assini-Meytin L, Casper ET, Fryer CS (2017). The influence of race and ethnicity on becoming a human subject: factors associated with participation in research. Contemp Clin Trials Commun.

[60] Kurupati R, Kossenkov A, Haut L, Kannan S, Xiang Z, Li Y (2016). Race-related differences in antibody responses to the inactivated influenza vaccine are linked to distinct pre-vaccination gene expression profiles in blood. Oncotarget..

[61] Quinn SC, Kumar S, Freimuth VS, Musa D, Casteneda-Angarita N, Kidwell K (2011). Racial disparities in exposure, susceptibility, and access to health care in the US H1N1 influenza pandemic. Am J Public Health.

[62] Browne Matthew, Thomson Patricia, Rockloff Matthew Justus, Pennycook Gordon (2015). Going against the Herd: Psychological and Cultural Factors Underlying the ‘Vaccination Confidence Gap’. PLOS ONE.

[63] Dubé E, Laberge C, Guay M, Bramadat P, Roy R, Bettinger JA (2013). Vaccine hesitancy. Hum Vaccin Immunother.

[64] Yaya S, Olarewaju O, Oladimeji KE, Bishwajit G (2019). Determinants of prenatal care use and HIV testing during pregnancy: a population-based, cross-sectional study of 7080 women of reproductive age in Mozambique. BMC Pregnancy and Childbirth.

[65] Yaya S, Bishwajit G (2019). Trends in the prevalence and care-seeking behaviour for acute respiratory infections among Ugandan infants. Global Health Research and Policy.

